# Neoadjuvant letrozole in postmenopausal estrogen and/or progesterone receptor positive breast cancer: A phase IIb/III trial to investigate optimal duration of preoperative endocrine therapy

**DOI:** 10.1186/1471-2407-8-62

**Published:** 2008-02-26

**Authors:** Ute E Krainick-Strobel, Werner Lichtenegger, Diethelm Wallwiener, Augustinus H Tulusan, Fritz Jänicke, Gunther Bastert, Ludwig Kiesel, Birgit Wackwitz, Stefan Paepke

**Affiliations:** 1 Department of Obstetrics and Gynecology, University of Tübingen, Calwerstraße 7, 72076 Tübingen, Germany; 2 Department of Obstetrics and Gynecology, Charité Medical University, Berlin, Germany; 3 Department of Obstetrics and Gynecology, Klinikum Bayreuth, Bayreuth, Germany; 4 Department of Gynecology, University Medical Center Hamburg-Eppendorf, Germany; 5 Department of Obstetrics and Gynecology, University of Heidelberg, Germany; 6 Department of Gynecology and Obstetrics, University of Münster, Germany; 7 Novartis Pharma GmbH, Nürnberg, Germany; 8 Women's Hospital, University Hospital Rechts der Isar, Technical University of Munich, Germany

## Abstract

**Background:**

In recent years, preoperative volume reduction of locally advanced breast cancers, resulting in higher rates of breast-conserving surgery (BCS), has become increasingly important also in postmenopausal women. Clinical interest has come to center on the third-generation nonsteroidal aromatase inhibitors (AIs), including letrozole, for such neoadjuvant endocrine treatment. This usually lasts 3–4 months and has been extended to up to 12 months, but optimal treatment duration has not been fully established.

**Methods:**

This study was designed as a multicenter, open-label, single-arm, exploratory phase IIb/III clinical trial of letrozole 2.5 mg, one tablet daily, for 4–8 months. The primary objective was to investigate the effect of neoadjuvant treatment duration on tumor regression and BCS eligibility to identify optimal treatment duration. Tumor regression (by clinical examination, mammography, and ultrasound), shift towards BCS eligibility, and safety assessments were the main outcome measures. Standard parametric and nonparametric descriptive statistics were performed.

**Results:**

Letrozole treatment was received by 32 of the enrolled 33 postmenopausal women (median (range): 67.0 (56–85) years) with unilateral, initially BCS-ineligible primary breast cancer (clinical stage ≥ T2, N0, M0). Letrozole treatment duration in the modified intent-to-treat (ITT; required 4 months' letrozole treatment) analysis population (29 patients) was 4 months in 14 patients and > 4 months in 15 patients. The respective per-protocol (PP) subgroup sizes were 14 and 11. The majority of partial or complete responses were observed at 4 months, though some beneficial responses occurred during prolonged letrozole treatment. Compared with baseline, median tumor size in the ITT population was reduced by 62.5% at Month 4 and by 70.0% at final study visit (Individual End). Similarly, in the PP population, respective reductions were 64.0% and 67.0%. Whereas initially all patients were mastectomy candidates, letrozole treatment enabled BCS (lumpectomy) in 22 ITT (75.9%) and 18 PP (72.0%) patients.

**Conclusion:**

Over half of patients become BCS-eligible within 4 months of preoperative letrozole treatment. While prolonged treatment for up to 8 months can result in further tumor volume reduction in some patients, there is no clear optimum for treatment duration. Letrozole has a favorable overall safety and tolerability profile.

**Trial registration:**

ClinicalTrials.gov identifier NCT00535418.

## Background

Until more recently, the conventional treatment of estrogen/progesterone receptor (ER/PgR) positive breast cancer in elderly postmenopausal women consisted primarily of surgery, followed by adjuvant endocrine or sequential chemo-endocrine therapy, and radiotherapy, depending on type of surgery (mastectomy or breast conservation), tumor volume and lymph node involvement [1]. In practice, regardless of disease stage, elderly patients still more frequently undergo mastectomy and receive less aggressive adjuvant treatment because of toxicity concerns; moreover they are also more likely to be given exclusively endocrine therapy if their tumors are hormone-sensitive [2].

Volume reduction of locally advanced breast cancers and the resulting rise in the rate of breast-conserving operations have come to play a progressively more important role also in elderly patients. Recent reports increasingly support the concept of primary endocrine therapy as an option for postmenopausal women with locally advanced receptor-positive breast cancer [3-6].

In the 1990s, the selective estrogen receptor modulator tamoxifen, until recently the gold standard in the adjuvant and metastatic treatment settings, was shown also to be effective as neoadjuvant endocrine therapy [7-9]. More recently, however, the focus of clinical interest has shifted to the third-generation nonsteroidal aromatase inhibitors (AIs) [10] such as letrozole and anastrozole as these drugs appear to produce at least comparable (anastrozole) or better overall response rates and permit more conservative subsequent surgery than tamoxifen [11-14].

Early neoadjuvant letrozole studies, in which postmenopausal patients were usually treated for 3 or 4 months [15,3,16], suggested that prolongation of AI treatment might further improve tumor shrinkage and down-staging, thus facilitating breast-conserving surgery (BCS). We therefore undertook the present study to investigate the potential benefits of extended neoadjuvant letrozole therapy with a view also to identifying optimal treatment duration.

## Methods

### Study design and setting

Conducted at six breast cancer treatment centers in Germany, this study was a multicenter, open-label, single-arm, exploratory phase IIb/III clinical trial of pre-operative letrozole treatment in postmenopausal women aged 55 years and older with untreated primary breast cancer. The neoadjuvant endocrine treatment regimen consisted of one 2.5 mg letrozole tablet daily for 4 to 8 months. The trial was approved in advance by, inter alia, the Ethics Committee of the Faculty of Medicine of the University of Tübingen and carried out in compliance with Good Clinical Practice guidelines (the 1964 Declaration of Helsinki as last amended in 1996; Directive 91/507/EEC; and US 21 Code of Federal Regulations, parts 50 and 56).

The primary study objective was to investigate the effect of the duration of treatment with letrozole 2.5 mg on tumor regression and on patient eligibility for breast-conserving surgery (BCS). Additional objectives included assessment of the safety and tolerability of the letrozole treatment regimen and subsequent procedures. All patients exposed to one or more doses of letrozole were included in the analysis of safety and tolerability (Safety).

The trial was originally designed to include ≥ 30 patients who, after showing clinical complete response (CR) or partial response (PR) at Month 4 (120 days, range 105–135 days), would continue letrozole treatment up to Month 8 (240 days, range 225–255 days).

### Patients

#### Inclusion criteria

Patients were required to be postmenopausal females (no spontaneous menses for ≥ 1 year; LH and FSH levels > 40 IU/L; or bilateral oophorectomy prior to breast cancer diagnosis) older than 55 years with primary invasive breast cancer, to give prior written informed consent and to be able to comply with the study protocol. Tumors had to be (a) of clinical stage T2 (only if ineligible for breast-conserving surgery (BCS)), T3, T4a, T4b, or T4c, in conjunction with N0 and M0 (no palpable axillary lymph nodes; no metastases); (b) measurable by clinical palpation, mammography (MG) and ultrasound (US); and (c) histologically confirmed by core needle biopsy and positive for estrogen receptors (ER) and/or progesterone receptors (PgR) as defined by core biopsy immunohistochemistry with > 30% positive malignant epithelial cells.

Additional requirements included adequate bone marrow function (white blood cell count (WBC) ≥ 3.5 × 10^9^/L; platelet count ≥ 100.0 × 10^9^/L) and hemoglobin levels (> 11.0 g/dL), adequate renal function (creatinine < 120 μmol/L) and hepatic function (bilirubin < 25 μmol/L, aspartate transaminase (AST) < 60 IU/L), and a life expectancy of at least six months.

#### Exclusion criteria

Patients were not included in the trial if they were eligible for breast-conserving surgery or had a history or diagnosis of (a) prior treatment with AIs or antiestrogens; (b) uncontrolled endocrine disorders such as diabetes, confirmed hypothyroidism or hyperthyroidism, Cushing's syndrome, or Addison's disease (treated or untreated); (c) unstable angina, uncontrolled cardiac disease; (d) bilateral breast tumors; (e) inflammatory breast cancer or distant metastasis; or (f) concurrent malignant disease (with the exception of cone-biopsied cervical carcinoma in situ, adequately treated basal or squamous cell carcinoma of the skin, or other curable cancers (e.g. Hodgkin's or non-Hodgkin's lymphomas), provided five recurrence-free years had elapsed since completion of therapy).

Also excluded from participation were patients with multifocal tumors (more than one lesion within any one quadrant of the breast) and patients receiving concomitant anti-cancer treatments such as chemotherapy, immunotherapy/biological response modifiers (BRMs), endocrine therapy (including steroids), bisphosphonate therapy (except for osteoporosis, in which case it was acceptable as concomitant therapy) and radiotherapy. Hormone replacement therapy (HRT) did not constitute an exclusion criterion provided it was discontinued at least two weeks before entry into the study. Concomitant treatment with steroids (e.g. glucocorticoids for indications other than cancer) was permitted only as aerosol treatment for obstructive airways disease or as intraarticular steroid injections for the treatment of inflammation. Patients who had received other investigational drugs within the preceding 30 days or were concomitantly taking other investigational drugs were also excluded from study participation.

### Treatments

#### Study drug

Letrozole (code name CGS 20267, trade name Femara^®^), 2.5 mg tablets) from a single batch (No. S05400) was supplied by Novartis Pharma GmbH, Nuremberg, Germany to the main investigator at each of the participating centers. Drug supplies were for use in this study only and had to be stored securely and in accordance with the storage conditions specified on the study drug labels.

#### Dose regimen

Patients took one oral dose (one 2.5 mg tablet) of letrozole a day for a period of up to 8 months. Study medication accounting was carried out by study center, but the study did not provide for a determination of compliance rates. Surgery was scheduled 4 months (105–135 days) from the date of first study treatment. In late-onset responders, surgery was postponed until Month 8 (225–255 days) if necessary and feasible to ensure there would be no interval between the last day of study treatment, and surgery. There was no reference treatment as this was an uncontrolled trial with only one treatment arm. Concomitant medication was permitted within the confines of the above exclusion criteria.

#### Tumor response assessments and study visits

Clinical tumor measurements and mammography and breast ultrasound examinations were performed at baseline, at monthly post-baseline visits – the visits at Month 4 (after 105–135 days) and Month 8 (after 225–255 days of letrozole treatment) being of central importance to the study – and at the patient's last study visit (Individual End) to determine clinical tumor response. CR was defined as the disappearance of all known disease, as determined by two observations obtained no less than 4 weeks apart. Similarly, PR was considered to have occurred if total tumor size decreased by at least 50% in the absence of any progression or new lesions. Decreases by < 50% and increases by < 25% were considered to represent no change (NC), while increases ≥ 25% or appearance of new lesions (were classed as progressive disease (PD).

### Statistical considerations and analysis

#### Sample size

No formal sample size estimation was performed for this exploratory trial. The targeted sample size was 30 patients who would continue letrozole treatment beyond Month 4. It was estimated that about 50 patients would need to be recruited, because 20% (10 patients) would experience treatment failure (with subsequent surgery) and another 20% (10 patients) would undergo surgery at Month 4 for other reasons.

#### Statistical analysis

Data analysis was performed by IMEREM GmbH, Nuremberg, Germany, on behalf of, and in close cooperation with, Novartis Pharma GmbH and in accordance with the study protocol, the statistical analysis plan as well as sponsor and IMEREM standard operating procedures. All statistical analysis was conducted at a descriptive level using Base SAS^® ^software (SAS Institute Inc., Cary, NC, U.S.A.), Version 8.2 for Windows^®^. No confirmatory hypothesis testing was planned or performed. In the case of statistical comparisons, results were interpreted in a descriptive and exploratory manner. Standard descriptive statistics were used, which comprised both parametric (including confidence intervals) and nonparametric methods.

##### Data presentation and analysis of baseline disease characteristics

Data were summarized using contingency tables for qualitative variables (n, %) and summary statistics for quantitative variables (mean and standard deviation; median and range).

##### Efficacy Analysis

First, tumor regression with treatment duration of at least 4 months was confirmed by calculating the response rates based on clinical tumor assessment, MG, and breast US. Second, regression analysis to determine the effect of treatment duration on tumor regression was performed for patients who were not considered treatment failures after 4 months of letrozole. The effects of baseline tumor size, nodal involvement, and age on treatment response were evaluated descriptively.

##### Safety analysis

This was based mainly on the types and frequencies of adverse events (AEs), which were classified by body system and preferred term and summarized as the number and percentage of patients experiencing at least one such AE. Additional information (e.g. severity or relationship to study drug) was presented in by-patient tabular listings.

#### Analysis populations

In addition to, and in modification of, the protocol-defined Safety and ITT populations, further analysis subpopulations were defined post hoc, as provided for in the protocol for this exploratory study. Thus data analysis was performed for the following three subsets of patients.

##### Safety population

This subset included all patients treated with at least one dose of study medication.

##### Intent-to-treat (ITT) population

In view of the primary objective of the trial, this efficacy population was modified a posteriori to exclude both untreated patients and those who took study medication for less than 4 months (< 105 days), i.e. the minimum treatment duration for clinically sound assessment of letrozole efficacy with respect to tumor shrinkage. Tumor assessment by at least one study assessment method (clinical assessment, breast US, or MG) after 4 months (105–135 days) of letrozole treatment was an additional ITT requirement.

##### Per-protocol (PP) population

Defined post hoc, the PP efficacy population was obtained by excluding from the ITT subset all patients with major protocol violations, these being defined as (a) an interval of more than 30 days between the last dose of letrozole and breast surgery; (b) the patient's refusal to undergo surgery; (c) deviation from clinically relevant selection criteria; and (d) any treatment with prohibited medication.

### Efficacy and safety assessments

The main outcome measures were tumor regression (as assessed by clinical examination, MG, and bidimensional breast US, and evaluated according to the WHO criteria [17]), shift towards BCS eligibility, and safety assessments. Any concomitant medication, coexisting diseases, and adverse events were coded using WHO dictionaries or a similar thesaurus before entry into the database.

**Efficacy assessments **included clinical assessment, MG, and breast US by the investigator. For each methodology, response status was based on WHO criteria. The results of tumor assessment by the investigators were used for statistical analysis.

**Safety assessments **consisted of monitoring and recording all adverse events, serious adverse events (with their severity and, if any, relationship to the study drug) and assessments of vital signs (systolic and diastolic blood pressure, and pulse rate), including body weight/body mass index (BMI). Adverse events (AEs) were recorded in the Case Report Form, stating duration, severity (Grade 1–4 (mild, moderate, severe or life-threatening)), relationship to the study drug (suspected/not suspected), and any action(s) taken. Serious AEs were recorded on a separate, purpose-designed form.

## Results

### Patients

#### Disposition, baseline demographics, and disease characteristics

The study enrolled 33 postmenopausal Caucasian women at 6 breast cancer centers in Germany between June 15, 2000 and July 23, 2002 (last completion). The number of patients per center ranged between 2 (3 centers) and 16 (1 center). Of those enrolled, 32 patients were exposed to at least one dose of letrozole, 3 discontinued treatment, and 29 completed letrozole treatment. The one patient who was not exposed was excluded from all analyses. Slow recruitment ultimately resulted in discontinuation of recruitment and premature termination of the trial.

Table 1 summarizes the baseline demographic data for the 32 patients who were evaluable for safety analysis.

**Table 1:** Demographic data at study entry for the Safety population of 32 postmenopausal Caucasian women with untreated primary breast cancer

	**Mean ± SD**	**Median**	**(Range)**
		
**Age **(yrs)	68.4 ± 7.2	67.0	(56–85)
**Weight **(kg)	72.6 ± 16.4	68.5	(49–120)
**BMI **(kg/m^2^)	27.2 ± 5.4	26.0	(20.1–44.6)

All patients were postmenopausal as defined by the inclusion criteria. Overall, the sample composition closely matched the targeted population. The disease characteristics are summarized in Table 2. The typical patient presented with breast cancer in the upper outer quadrant, the tumor being moderately differentiated and predominantly of ductal histology. All 32 patients exposed to letrozole had primary tumors that tested positive for estrogen and/or progesterone receptors (ER+/PgR+). One patient was positive for progesterone receptor only. The proposed surgery at baseline was mastectomy for all patients (information missing for one patient).

**Table 2:** Diagnosis and extent of cancer in the Safety population (N = 32) at study entry

	**No. of patients**	**% of Safety population**
		
**Clinical tumor categories**		
T2*	23	71.9
T3	5	15.6
T4a	1	3.1
T4b	3	9.4
**Primary tumor histology**		
Ductal	19	59.4
Invasive lobular/variant	8/1	25.0/3.1
Other/missing	3/1	9.4/3.1
**Tumor grade**		
Well/moderately/poorly differentiated	3/16/2	9.4/50.0/6.3
Missing	11	34.4
**Positive receptor status**		
Estrogen/progesterone/either	31/25/32	96.9/78.1/100.0

* only if ineligible for breast-conserving surgery

#### Patient exposure

The duration of letrozole treatment (N = 32) ranged from 2 to 8 months. The main treatment duration categories with 3 or more patients were 4, 5, and 8 months, which comprised 14, 5, and 7 (43.8, 15.6, and 21.9%), respectively, i.e. 81.3% in total, of the 32 Safety-evaluable patients. Median total duration of treatment was about 130 days (4.3 months) with a range from 56 to 258 days (1.9–8.5 months), as detailed in Table 3). Of the 15 patients treated for more than four months, 7 (i.e. 21.9% of the Safety population, or 24.1 and 28% of the ITT and PP populations, respectively (cf. Table 4)) received letrozole for about 8 months.

**Table 3:** Overall treatment duration in the Safety population (N = 32)

	**Months of treatment**			
	**< 4**	**4**	**> 4**	**Total**
		
No. of patients	3	14	15	32
Median duration (range) (in days)	65 (56–94)	121 (105–130)	214 (138–258)	129.5 (56–258)
Mean duration ± SD (in days)	71.7 ± 19.9	120.1 ± 7.1	200.9 ± 46.4	153.5 ± 57.1

**Table 4:** Number of patients (and percentage of subgroup total) in the Safety and Efficacy (ITT and PP) analysis populations, grouped by treatment duration

	**Treatment duration (months)**			
**Population**	**< 4**	**4**	**> 4**	**Total**
		
Safety	3 (9.4%)	14 (43.8%)	15 (46.9%)	32
Intent-to-treat (ITT)	n.a.	14 (48.3%)	15 (51.7%)	29
Per-protocol (PP)	n.a.	14 (56.0%)	11 (44.0%)	25

n.a. = not applicable as this analysis population, by definition, contained no patients treated for less than 4 months

#### Analysis populations

The sizes of the three analysis populations in this study are given in Table 4, together with the sizes of subgroups by treatment duration.

The final number of patients enrolled was considered sufficient to allow descriptive analysis of the safety (32 safety-evaluable patients) and efficacy of letrozole and to estimate the potential benefits of preoperative letrozole as a neoadjuvant treatment in postmenopausal primary breast cancer.

### Effect of treatment duration on tumor regression

#### Tumor response

The WHO tumor response results at Month 4, Month 8 and Individual End of treatment (final examination) are summarized for the ITT and PP populations in Table 5.

**Table 5:** WHO tumor response status (n, %) in the ITT and PP populations at Months 4 and 8 and Individual End of letrozole treatment

	**Intent-to-treat population, N = 29**						**Per-protocol population, N = 25**					
	**Month 4**		**Month 8**		**Individual End**		**Month 4**		**Month 8**		**Individual End**	
		
**Tumor response**	**Clinical tumor assessment**						**Clinical tumor assessment**					
complete response	1	*(3.5%)*	1	*(3.5%)*	2	*(6.9%)*	1	*(4.0%)*	1	*(4.0%)*	2	*(8.0%)*
partial response	15	*(51.7%)*	6	*(20.7%)*	19	*(65.5%)*	14	*(56.0%)*	3	*(12.0%)*	16	*(64.0%)*
no change (stable disease)	5	*(17.2%)*	1	*(3.5%)*	4	*(13.8%)*	3	*(12.0%)*	0	*(0.0%)*	3	*(12.0%)*
progressive disease	2	*(6.9%)*	0	*(0.0%)*	3	*(10.3%)*	2	*(8.0%)*	0	*(0.0%)*	3	*(12.0%)*
Missing observations	6	*(20.7%)*	21	*(72.4%)*	1	*(3.5%)*	5	*(20.0%)*	21	*(84.0%)*	1	*(4.0%)*
		
**Tumor response**	**Mammography**						**Mammography**					
complete response	0	*(0.0%)*	0	*(0.0%)*	0	*(0.0%)*	0	*(0.0%)*	0	*(0.0%)*	0	*(0.0%)*
partial response	7	*(24.1%)*	2	*(6.9%)*	7	*(24.1%)*	5	*(20.0%)*	1	*(4.0%)*	5	*(20.0%)*
no change (stable disease)	11	*(37.9%)*	1	*(3.5%)*	13	*(44.8%)*	10	*(40.0%)*	0	*(0.0%)*	11	*(44.0%)*
progressive disease	1	*(3.5%)*	0	*(0.0%)*	1	*(3.5%)*	1	*(4.0%)*	0	*(0.0%)*	1	*(4.0%)*
Missing observations	10	*(34.5%)*	26	*(89.7%)*	8	*(27.6%)*	9	*(36.0%)*	24	*(96.0%)*	8	*(32.0%)*
		
**Tumor response**	**Breast ultrasound**						**Breast ultrasound**					
complete response	1	*(3.5%)*	1	*(3.5%)*	2	*(6.9%)*	1	*(4.0%)*	1	*(4.0%)*	2	*(8.0%)*
partial response	13	*(44.8%)*	5	*(17.2%)*	16	*(55.2%)*	11	*(44.0%)*	2	*(8.0%)*	13	*(52.0%)*
no change (stable disease)	6	*(20.7%)*	1	*(3.5%)*	8	*(27.6%)*	5	*(20.0%)*	1	*(4.0%)*	7	*(28.0%)*
progressive disease	1	*(3.5%)*	0	*(0.0%)*	1	*(3.5%)*	1	*(4.0%)*	0	*(0.0%)*	1	*(4.0%)*
Missing observations	8	*(27.6%)*	22	*(75.9%)*	2	*(6.9%)*	7	*(28.0%)*	21	*(84.0%)*	2	*(8.0%)*

#### Comparison of tumor assessments

Overall, clinical tumor assessment at Individual End yielded the highest response rates (complete or partial response: 21/29 (72.4%) ITT patients; 18/25 (72.0%) PP patients). MG at Individual End yielded the least favorable findings (no complete, only partial responses in 7/29 (24.1%) ITT patients and 5/25 (20.0%) PP patients). Results for breast US at Individual End were somewhat less favorable compared with the clinical tumor assessments (complete or partial response in 18/29 (62.1%) ITT patients and 15/25 (60.0%) PP patients).

#### Treatment duration and tumor response

Of the 5 ITT patients without change in **clinical tumor assessment **at Month 4, 2 showed partial response and 1 complete response at Month 8. None of the patients with unchanged **MG **findings at Month 4 showed a more favorable response at Month 8, but 1 patient whose breast **US **was unchanged at Month 4 had partial response at Month 8. In the PP population, responses were observed in 9 of 24 (37.5%) evaluable patients at Month 4 without additional treatment, and in another 9 patients after extended treatment, 6 patients having had partial or complete response at Month 4. Thus, partial or complete response was already achieved in 15 patients (62.5%) after 4 months of letrozole treatment.

#### Reduction in tumor size

Compared with baseline, median tumor size in the ITT population was reduced by 62.5% at Month 4 and by 70.0% at Individual End. In the PP population, median tumor size was similarly reduced by 64.0% (Month 4) and 67.0% (Individual End).

#### Relationship between treatment duration and tumor response

Table 6 shows the results for the ITT and PP populations of the threefold statistical analysis of clinical tumor assessment, MG, and breast US as calculated by Pearson correlation (percent change in tumor size from baseline to Individual End vs. days on letrozole treatment), Spearman correlation (tumor response categories 1 (CR) to 4 (PD)) at Individual End vs. days on letrozole treatment), and multiple regression analysis (percent change in tumor size as criterion, and days on treatment, age, and tumor size at baseline as predictors).

**Table 6:** Relationship between treatment duration and tumor response

	**Clinical assessment**		**Mammography**		**Breast ultrasound**	
	
	ITT (n = 28)	PP (n = 24)	ITT (n = 21)	PP (n = 17)	ITT (n = 27)	PP (n = 23)
**Pearson correlation**	r = -0.3146	r = -0.4003	r = -0.2396	r = -0.2956	r = -0.2099	r = -0.3188
	p = .1030	p = .0526	p = .2955	p = .2494	p = .2934	p = .1382

**Spearman correlation**	r = -0.3839	r = -0.5092	r = -0.1037	r = +0.0409	r = -0.4111	r = -0.3371
	p = .0437	p = .0110	p = .6548	p = .8761	p = .0331	p = .1158

**Multiple regression analysis**	F = 1.96	F = 1.66	F = 0.87	F = 0.60	F = 0.73	F = 1.05
	p = 0.1471	p = 0.2070	p = 0.4748	p = 0.6278	p = 0.5465	p = 0.3928
	R^2 ^= 0.1967	R^2 ^= 0.1996	R^2 ^= 0.1334	R^2 ^= 0.1212	R^2 ^= 0.0866	R^2 ^= 0.1424
	R^2^* = 0.0963	R^2^* = 0.0795	R^2^* = -0.0195	R^2^* = -0.0816	R^2^* = -0.0326	R^2^* = 0.0070

* adjusted.

Threefold statistical analysis performed for clinical tumor assessment, MG, and breast US included: a) Pearson correlation of percent change in tumor size from baseline to Individual End vs. days on letrozole treatment; b) Spearman correlation of tumor response categories (1 = CR to 4 = PD) at Individual End vs. days on letrozole treatment; and c) multiple regression analysis of percent change in tumor size as criterion vs. days on treatment, age, and tumor size at baseline as predictors (3 degrees of freedom). Deviations in ITT and PP population sizes from 29 and 25, respectively, were due to non-analyzable patients.

As regards clinical tumor assessment, there was a moderate correlation between the clinically assessed tumor response at Individual End and treatment duration in that tumor size decreased and calculated tumor response improved with increasing treatment duration.

The correlations between treatment duration and decrease in tumor size were slightly higher in the PP population than in the ITT population, indicating that letrozole had a favorable effect on tumor shrinkage when treatment was administered as planned, i.e. for a sufficiently long period. The variables examined in the multiple regression analyses failed to explain a significant portion of the variance in the percent change in tumor size observed between baseline and Individual End of letrozole treatment.

For clinical tumor assessment, there was a moderate Pearson correlation between treatment duration and change in tumor size from baseline (ITT: r = -0.3146; PP: r = -0.4003), and a substantial Spearman correlation between treatment duration and tumor response (ITT: r = -0.3839; PP: r = -0.5092). The respective MG correlations were modest or negligible (Pearson, ITT: r = -0.2396; PP: r = -0.2956; Spearman, ITT: r = -0.1037; PP: r = +0.0409), and the breast US correlations were low to moderate (Pearson, ITT: r = -0.2099; PP: r = -0.3188; Spearman, ITT: r = -0.4111; PP: r = -0.3371), with substantial negative correlations indicating that longer treatment duration was associated with a more favorable response. Multiple regression analysis revealed no consistent and statistically relevant influence of age and tumor size at baseline.

#### Tumor staging, lymph nodes, and metastases

Changes in pathological tumor staging are given in Table 7 for all patients exposed to study drug, including those treated less than 4 months. Overall, the changes were observed after a median treatment duration of 4.3 months. The data demonstrate that a shift to less severe T-stages occurred in the course of treatment. Moreover, at the final assessment, no or only very few lymph nodes were affected in the majority of patients and none of the 30/32 patients examined had metastases (data not shown).

**Table 7:** Tumor staging before and after letrozole treatment (Safety population (N = 32), number and percentage of patients)

**Clinical stage**	**Baseline**	**Individual End**
T1a	--	1 (3.1%)
T1b	--	1 (3.1%)
T1c	--	7 (21.9%)
T2	23 (71.9%)	19 (59.4%)
T3	5 (15.6%)	--
T4a	1 (3.1%)	--
T4b	3 (9.4%)	--
Missing	--	4 (12.5%)

#### Subsequent breast surgery

The lumpectomy and mastectomy frequencies in the Safety and Efficacy populations are summarized in Table 8. All patients treated with letrozole for 4 months underwent surgery no later than 30 days after taking the last dose of study drug. Of the patients treated for more than 4 months, the majority (73.3%) of the ITT population and all (100%) PP patients had breast surgery within 30 days of the last dose of study medication. Whereas initially all patients were candidates for mastectomy, letrozole treatment enabled BCS (lumpectomy) in 22/29 (75.9%) ITT patients and 18/25 (72.0%) PP patients.

**Table 8:** Lumpectomy and mastectomy frequencies in the Safety and Efficacy populations after study treatment (number of patients and percentage of respective analysis subset)

	**Safety**	**ITT**		**PP**	
	
	**N = 32**	**= 4 months (N = 14)**	**> 4 months (N = 15)**	**= 4 months (N = 14)**	**> 4 months (N = 11)**
	
	n *(%)*	n *(%)*	n *(%)*	n *(%)*	n *(%)*
Lumpectomy	22 *(68.8%)*	10 *(71.4%)*	12 *(80.0%)*	10 *(71.4%)*	8 *(72.7%)*
Mastectomy	9 *(28.1%)*	4 *(28.6%)*	3 *(20.0%)*	4 *(28.6%)*	3 *(27.3%)*
Missing data	1 *(3.1%)*	--	--	--	--
Total	32 *(100.0%)*	14 *(100.0%)*	15 *(100.0%)*	14 *(100.0%)*	11 *(100.0%)*

### Safety and tolerability

#### Adverse events

Of the 82 adverse events (AEs; MedDRA low level terms, no multiple counts) reported in 27/32 (84.4%) Safety-evaluable patients, only 1 AE was both severe and serious (colon cancer, *nos *(not otherwise specified)). One other serious adverse event (SAE) that occurred in a different patient (upper limb fracture *nos*) was moderate but required hospitalization. Neither SAE was suspected to be related to letrozole. None of the non-serious AEs led to discontinuation, temporary interruption of treatment with study drug, or dose adjustment. No deaths were reported in this study.

#### Other safety assessments

No laboratory assessments were performed. Vital signs (diastolic and systolic blood pressure, pulse rate, and body weight/body mass index), some abnormal at baseline, generally improved rather than deteriorated over the course of the study.

## Discussion

The third-generation AIs are increasingly challenging tamoxifen, hitherto the gold standard in the endocrine treatment of estrogen-responsive postmenopausal breast cancer, in both the neoadjuvant and adjuvant settings [18]. For instance, superior efficacy of letrozole over tamoxifen as neoadjuvant treatment for postmenopausal patients with ER+/PgR+ breast cancer has been demonstrated by Eiermann et al. [3]. More recently, Paepke et al. [19] and Renshaw et al. [20] published abstracts of their findings regarding the optimal duration of neoadjuvant treatment with letrozole. To date, no other study has addressed this issue. The current paper is the first full report of the data previously presented in brief by Paepke and coworkers.

The present study was conducted as a single-arm exploratory trial primarily to investigate the effect of treatment duration with letrozole 2.5 mg/day on tumor regression and eligibility for breast-conserving surgery (BCS) in postmenopausal primary hormone-responsive breast cancer, also with a view to identifying optimum duration of treatment. The trial was stopped prematurely due to slow patient recruitment at the participating centers. Therefore, instead of the planned total number of 50 patients, the number of Safety-evaluable patients recruited was 32, with 29 and 25 patients being available for the ITT and PP analyses, respectively, and 15 ITT and 11 PP patients instead of the expected 30 patients receiving more than four months' letrozole treatment. In deviation from the study protocol, letrozole treatment was prolonged in four patients who showed no change as defined by the WHO criteria (i.e. had stable disease) at the Month 4 assessment. In these cases, the investigators' clinical judgment and expectation that patients would benefit from further letrozole treatment was given priority over strict adherence to the protocol schedule, with the result that three of the four patients benefited from prolonged letrozole therapy. Overall, however, the down-sizing of the study and the minor deviations from the study protocol were considered acceptable in view of the exploratory nature of the trial.

The definition of the intent-to-treat (ITT) population used in this study was a stricter modification of the common definition in that it excluded 3 patients who received less than 4 months (105 days) of letrozole treatment. However, to enable analysis in accordance with the primary study objective, this restriction was considered necessary since a clinically valid assessment of letrozole efficacy in terms of tumor shrinkage required a minimum of 4 months' treatment. An additional ITT requirement was a final examination using at least one of the three study assessment methods, i.e. clinical palpation, MG, or breast US.

Under the conditions of our study, the majority of patients responded to letrozole treatment by Month 4, with respective overall ITT and PP response rates of 55.2% (CR 3.5%, PR 51.7%) and 60% (CR 4.0%, PR 56.0%) for clinical palpation. Of the 15 ITT and 11 PP patients treated until Month 8, 7 (1 CR, 6 PR) and 4 (1 CR, 3 PR) patients, respectively, showed response as determined by clinical palpation, yielding overall response rates of 24.2% (CR 3.5%, PR 20.7%) and 16.0% (CR 4.0%, PR 12.0%) relative to the respective overall population sizes of 29 and 25, and thus indicating benefit from prolonged letrozole treatment. At Individual End, reflecting general therapeutic benefit from 4 to 8 months' neoadjuvant oral letrozole, respective overall clinical palpation response rates in ITT and PP patients were 72.4% (CR 6.9%, PR 65.5%) and 72.0% (CR 8.0%, PR 64.0%), suggesting incremental benefit from letrozole treatment of more than 4 months' duration.

For all assessment times, overall response rates were generally similar for clinical palpation and breast US but markedly less favorable for MG. The concordance between assessment methods, or lack thereof, awaits further investigation.

Our finding that median tumor size was reduced by 70% and 67.0% at Individual End vs. 62.5% and 64.0% at Month 4 in the ITT and PP populations, respectively, lends further support to the conclusion that prolongation of letrozole treatment can be associated with incremental therapeutic benefit.

Similarly, correlation analyses of treatment duration versus change in tumor size from baseline (Pearson) and tumor response (Spearman) as assessed by clinical palpation yielded moderate and substantial correlations, respectively, while multiple regression analysis revealed no consistent, statistically relevant influence of age and baseline tumor size on percent change in tumor size.

Over the course of treatment, there was a demonstrable overall downward shift in tumor stages from an initial T2-T4b range to T1a-T2 at Individual End of letrozole treatment. As regards subsequent breast-conserving surgery in our study, neoadjuvant letrozole treatment enabled lumpectomy to be performed in 10/14 (71.4%) ITT and 10/14 (71.4%) PP patients after 4 months' treatment and in 12/15 (80.0%) ITT and 8/11 (72.7%) PP patients treated for more than 4 months. This underlines the beneficial effect of neoadjuvant letrozole treatment for 4 months or longer.

The observed adverse events considered to be related to the study medication were not unexpected and mostly mild to moderate in severity. Two severe adverse events were classified as unrelated to the study drug. There were no deaths during the study period, and no dose adjustment or interruption of letrozole treatment was necessary. Hence, letrozole was generally very well tolerated and proved safe in this study.

As far as comparison is possible, our data appear to be in general agreement with findings so far available only as an abstract by Renshaw and coworkers [20], who carried out a prospective audit to assess response to 3–12 months' neoadjuvant treatment with letrozole 2.5 mg/day in 142 postmenopausal women with large operable or locally advanced ER-rich (Allred score ≥ 6) breast cancer. The investigators concluded that neoadjuvant letrozole produced ongoing tumor shrinkage over periods up to 12 months in patients responding to letrozole at 3 Months, but that there was no optimum duration for the use of neoadjuvant letrozole.

## Conclusion

Based on our findings we draw the overall conclusion that neoadjuvant letrozole treatment renders more than half of postmenopausal ER+/PgR+ mastectomy candidates eligible for lumpectomy within only 4 months and that prolonged treatment for up to 8 months can result in further tumor volume reduction and, thus, provide incremental benefit to patients. Our exploratory data do not suggest that there is an optimum duration for the preoperative use of letrozole. However, our study confirms that letrozole possesses a very favorable toxicity profile and can be safely and effectively used for at least 8 months in the neoadjuvant setting.

## Abbreviations

AE = adverse event; AI = aromatase inhibitor; AST = aspartate transaminase; BCS = breast-conserving surgery; BMI = Body Mass Index; BP = blood pressure; BRM = biological response modifier; CR = complete response; EEC = European Economic Community; ER = estrogen receptor; ER+ = ER positive; HRT = hormone replacement therapy; ITT = intent-to-treat; MedDRA = Medical Dictionary for Regulatory Affairs; MG = mammography; NC = no change (stable disease); *nos *= not otherwise specified (refers to MedDRA); PD = progressive disease; PgR = progesterone receptor; PgR+ = PgR positive; PP = per-protocol ; PR = partial response; SAE = serious adverse event; US = ultrasound; WBC = white blood cells; WHO = World Health Organization

## Competing interests

The study sponsor was Novartis Pharma GmbH, Nuremberg, Germany. BW is an employee of Novartis Pharma GmbH, Nuremberg, Germany. The other authors declare they have no competing interests.

## Authors' contributions

UKS, WL, DW, AHT, FJ, GB, LK, and SP made substantial contributions to the conduct of the study, the treatment of patients, and the collection of data. UKS contributed to the drafting and revision of the manuscript. BW participated in the study design and coordination and made contributions to data collection, analysis, interpretation and reporting, and to manuscript revision. All authors read and approved the final manuscript.

## Pre-publication history

The pre-publication history for this paper can be accessed here:


